# Prevalence of Near-Vision-Related Symptoms in a University Population

**DOI:** 10.3390/vision8020038

**Published:** 2024-06-19

**Authors:** Jessica Gomes, Sandra Franco

**Affiliations:** Centre of Physics of the Universities of Minho and Porto, University of Minho, 4710-057 Braga, Portugal; sfranco@fisica.uminho.pt

**Keywords:** symptomatology, near-vision, ocular accommodation

## Abstract

The university population has high visual demands. It is therefore important to assess the prevalence of symptoms in these subjects, which may affect their academic performance. In this cross-sectional study, a randomized sample of 252 subjects from a university answered the Convergence Insufficiency Symptom Survey (CISS) questionnaire. In addition, questions were asked about blurred vision during and after near tasks, the number of hours per day spent in near vision, and whether or not they wore glasses. Furthermore, 110 subjects underwent an eye exam, including a refraction and accommodation assessment. The mean age of the subjects was 28.79 ± 11.36 years, 62.3% reported wearing glasses, and on average 7.20 ± 2.92 hours/day was spent in near vision. The mean of the CISS score was 18.69 ± 9.96, and according to its criteria, 38% of the subjects were symptomatic. Some symptoms were significantly (*p* < 0.05) more frequent in subjects wearing glasses. Accommodative dysfunctions were present in 30.9% of the subjects, the most common being insufficiency of accommodation. We emphasise the importance of assessing symptomatology during the clinical examination in this group of subjects, as they spend many hours a day in near vision, as well as assessing accommodation, binocular vision, and the ergonomic work environment, which may be at the origin of the symptoms, in addition to the need to wear glasses.

## 1. Introduction

Excessive use of near vision has become increasingly prevalent in modern society due to the widespread adoption of digital devices, such as computers, tablets and smartphones, both at work and as entertainment. Doing these activities for excessive amounts of time may cause a range of symptoms, such as headache, visual strain, and red eyes, among others [1,2,3,4]. According to previous studies, up to approximately 90% of individuals who use digital devices for extended periods report experiencing eyestrain [1,5].

It has been suggested that near-vision tasks may contribute to the development of accommodative dysfunctions [6]. Previous studies showed that the use of digital screens for more than 20 minutes leads to a reduction in the amplitude of accommodation and accommodative facility, and increases accommodative lag [7,8,9]. These dysfunctions affect up to 61.7% [10] of the general population. Accommodative insufficiency has a prevalence between 2% and 61.7%, accommodative infacility between 0.4% and 5%, and accommodative excess between 1.8 % and 10.8% [11]. The wide range in the prevalence of accommodative dysfunctions suggests that this prevalence might depend on different factors, which may be related to the activities or jobs the subjects do. In addition, study methodologies, populations, and diagnostic criteria used may also contribute to this wide variation.

School, university or work take up almost one-third of the day for many people, and most of that time is spent reading, writing and using electronic devices. In the academic environment, individuals have significantly high demands on their visual system, performing detailed tasks, and for prolonged periods of time. In addition, visual acuity demand in the classroom has, in general, increased in recent years, both at far and near distances [12,13,14]. Therefore, high levels of visual acuity, contrast sensitivity, and good accommodation and binocular vision are needed to meet the requirements of modern university environments.

The integrity of visual skills is critical for success in studies or work, as 80% of learning is through the eyes [15]. Therefore, it is important to assess the prevalence of symptoms in this group of subjects, as their academic performance may be affected. Difficulties in maintaining clear and comfortable vision can lead to increased fatigue, reduced attention and reduced productivity [16,17].

There are few studies assessing the symptomatology associated with near-vision tasks in academic populations. Furthermore, few studies [18,19] reported the prevalence of accommodative dysfunctions in this population and only included students, finding a high prevalence, with 10.8% having excess of accommodation [19].

Previous research suggests that convergence and divergence insufficiency are associated with refractive error, with moderate and high myopes having a higher prevalence of divergence insufficiency and a lower prevalence of convergence insufficiency. Regarding accommodative insufficiency and infacility, no statistically significant differences were found between myopes and emmetropes [20]. Vergence dysfunctions were also associated with myopia progression, with children with convergence excess having greater myopia progression [21]. However, these studies did not include hyperopes, nor did they consider all accommodative and vergence dysfunctions.

This study aimed to assess near-vision-related symptoms in a university population and to determine the prevalence of accommodative dysfunctions in this population, and by refraction error.

## 2. Materials and Methods

This study was carried out in the academic population of the University of Minho (Braga, Portugal), including students, professors, and researchers. Individuals with a history of systemic or ocular disease or taking medication that could affect vision were not included in the study.

These subjects were required to complete the Convergence Insufficiency Symptom Survey (CISS). This is a validated questionnaire that includes 15 questions about visual symptoms during near-vision tasks and is widely used by researchers and clinicians. Each question has five possible responses, and each response is scored on a scale of 0 to 4, where 4 corresponds to the highest frequency of occurrence of symptoms (“always”) and 0 corresponds to “never”. The CISS score was calculated by summing the scores for each response [22]. Two additional questions related to symptomatology were also added to the questionnaire:

1. Do you notice the objects/words blurred at far distances after reading/doing close work?
2. Do you find it difficult to keep the objects/words clear when you alter the vision from far to near and/or near to far?

The subjects were also asked about the number of hours a day spent reading/doing close work and whether they use glasses or not. 

After the questionnaire, non-presbyopic subjects were invited for the second part of the study, including an eye exam consisting of:

1. Objective ocular refraction with static retinoscopy
2. Subjective ocular refraction started from the value of the objective refraction, and the maximum plus refraction for best visual acuity was found for each eye. Myopia was defined as a spherical equivalent refractive error of greater than or equal to −0.50 D and hyperopia greater than or equal to +0.50 D [23].
3. Assessment of monocular amplitude of accommodation by the minus lens method, adding negative lenses in steps of 0.25 D until the subject sees one line blur before the maximum visual acuity placed at 40 cm.
4. Assessment of lag of accommodation by monocular estimated method (MEM) retinoscopy. While the subject read the words of a MEM card, retinoscopy was performed along the horizontal axis and estimated the amount of plus or minus necessary to neutralize the motion of the reflex observed through the retinoscope.
5. Assessment of monocular facility of accommodation at near vision (targeting one line before the maximum visual acuity placed at 40 cm) with flippers of ±2.00 D.
6. Measurement of horizontal phoria using the von Graefe technique for distance and near vision. A phoropter with Risley prisms of 12 base-in and 6 base-out was used for distance vision, and 15 base-in and 9 base-out for near vision. A column of letters with a visual acuity of 0.8 was used as a fixation target at 6 m and 40 cm for the far and near measurement of phoria, respectively. Negative values represent exophoria, and positive values esophoria.
7. Measurement of positive and negative fusional vergences for far and near vision. The same target was used, and the values of blur, break and recovery were recorded.

Accommodative and binocular dysfunctions were diagnosed according to the criteria previously reported by Lara et al. [24] (Table 1).

Statistical analysis was performed using the software SPSS (Statistical Package for Social Sciences, version 29). The normality of the data was tested using the Kolmogorov–Smirnov (K-S) test. To compare the mean values, a *t*-test was used for data following a normal distribution, and the Mann–Whitney for a non-normal distribution. To analyse the correlation between data, the Pearson correlation coefficient was used for quantitative normally distributed variables; otherwise, Spearman’s correlation coefficient was used.

The study adhered to the tenets of the Declaration of Helsinki and was approved by the Ethical Sub Commission of Life and Health Science of the University of Minho. All subjects signed an informed consent with an explanation of the procedures.

## 3. Results

Two hundred and fifty-two subjects with a mean age of 28.8 ± 11.4 years answered the questionnaire. They reported spending on average 7.2 ± 2.9 hours a day reading or doing close work, and 157 subjects reported wearing glasses (62.3%).

The CISS score was calculated according to the criteria of the survey [25], obtaining a mean value of 18.7 ± 9.9. Considering symptomatic being a score of 21 or higher, 38% of the subjects were symptomatic and 62% were asymptomatic.

The percentages of the answers of all subjects for each question of the CISS and the two additional questions are shown in Table 2. The most common symptom reported by the subjects was feeling tired eyes when reading/doing close work, with 68.7% having this symptom sometimes, fairly often or always while reading/doing close work. The two added symptoms had high prevalence, showing the highest percentages of the answer “always”. The least common symptom was seeing words moving, jumping or seeming to float across the page when reading/doing close work.

The total CISS score did not show a significant correlation with age, and if we divide the subjects into non-presbyopic (214 subjects) and presbyopic (39 subjects) groups, the scores are, respectively, 18.5 ± 9.64 and 19.6 ± 11.6, without statistically significant differences between them (*p* = 0.23). However, an association between some symptoms and age was found. Older subjects were more likely than younger subjects to feel uncomfortable (*p* < 0.01) and to have red eyes (*p* = 0.02) when reading or doing close work, whereas trouble remembering (*p* < 0.01) and the need to reread the same line (*p* = 0.01) is more frequent in younger subjects.

**Figure 1:**
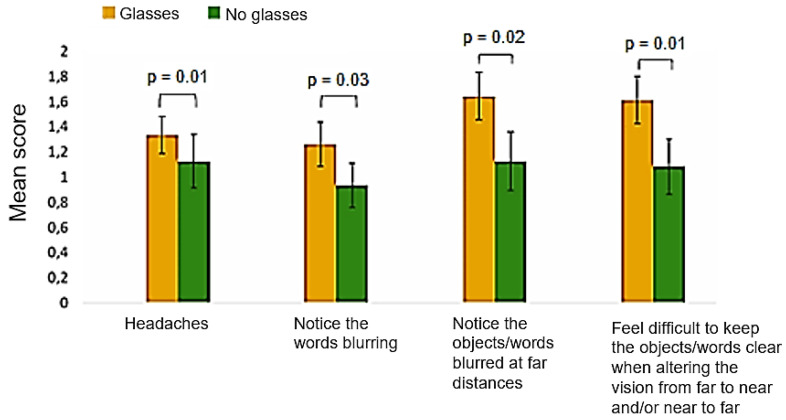
Mean score of the symptoms with statistically significant differences between subjects who wear and do not wear glasses. Error bars indicate the standard deviation.

A statistically significant association between some symptoms (headache; noticing the words blurring while reading/doing close work; noticing the objects/words blurred at far distances after reading/doing close work; and finding it difficult to keep the objects/words clear when altering the vision from far to near and/near to far) and wearing glasses was found, showing scores significantly higher for subjects who wear glasses than those who do not wear them. These differences are shown in Figure 1.

One hundred and ten non-presbyopic subjects with a mean age of 24.4 ± 5.0 years old underwent an eye exam. On average, the spherical equivalent determined by subjective refraction was −0.50 D ± 1.67 D, and the mean astigmatism was −0.42 D ± 0.43 D. Compared to the habitual refraction of the subjects who wore spectacles, 15.2% of them were overcorrected and 9.5% were undercorrected. Of those who were overcorrected, 87.5% were myopes who wore more negative refraction than they actually needed, and of those who were undercorrected, 20% were myopes who wore less than they needed. For astigmatism, the maximum discrepancy between the subject’s habitual refraction and the refraction we measured was 0.50 D, and it was found in 10% of the subjects.

The amplitude of accommodation was 8.77 D ± 2.35 D, the lag of accommodation was 0.65 D ± 0.50 D, and the facility of accommodation was 10.72 ± 6.21 cpm (cycles per minute). The amplitude of accommodation was negatively correlated with the accommodative lag (cor = −0.41, *p* < 0.01) and positively correlated with the facility of accommodation (cor = 0.41, *p* < 0.01).

The prevalence of accommodative dysfunctions is shown in Table 3, divided into infacility, excess, and insufficiency of accommodation. The most common was insufficiency of accommodation. None of these accommodative dysfunctions were previously diagnosed.

The mean values of phoria and the negative and positive fusional vergences for far and near vision are shown in Table 4. With regard to binocular dysfunctions, 1.82% had convergence insufficiency and 1.82% had convergence excess. No other binocular dysfunction was found.

The score of CISS and the number of hours spent in near vision was not significantly different between the subjects with (score = 16.6; hours/day = 7.1) and without (score = 17.8; h/day = 7.7) accommodative dysfunctions. However, 40% of the subjects analysed were symptomatic, according to the criteria described above, and from these symptomatic subjects, 39.4% had an accommodative dysfunction.

The prevalence of accommodative dysfunctions by refractive state was also analysed, and the results are shown in Table 5. Hyperopes seemed to have a higher prevalence of accommodative dysfunctions, whereas myopes had the lowest prevalence; however, this tendency is not statistically significant (*p* = 0.18). Due to the low prevalence of binocular dysfunctions, their prevalence by refractive errors was not reported.

## 4. Discussion

Knowledge of the symptomatology of the academic population will help to better manage the eye examination of these patients in clinical practice. In this study, we observed that these populations spend considerable time performing near-vision tasks (7.20 ± 2.92). This may lead to symptoms and vision problems, such as accommodative dysfunctions. On the other hand, vision problems already present may be felt with more intensity, as these subjects use their near vision excessively.

A high prevalence of symptoms was found (CISS score = 18.7 ± 9.9), with a high percentage of subjects reporting having these symptoms at some point during reading/doing close work. The CISS score was higher compared to the general adult population. A previous study in adults found a mean CISS score of 15.4 (range 0–40), with 24.5% having a score ≥21 [26]. Another recent study also revealed that 26% of adults had a score >21, a lower percentage than in our study (38%) [27]. As referred to above, since the university population tends to spend more time doing near-vision tasks due to work and study demands, it is expected that they will develop more symptoms related to this, or that they will notice more of these symptoms. A previous study with university students [25] also found a lower score of 15.2 ± 10.0, with 28% being symptomatic. In our study, having tired eyes was the symptom reported with the most frequency, and noticing objects/words blurred at far distances after reading/doing close work, and difficulty keeping objects clear when altering the vision from far to near and/or near to far, were the symptoms with the highest percentage for the answer “always”. This indicates a loss of accuracy in the accommodative system. A previous study among university students found digital eye strain in 68.53% of the subjects, but the most common symptoms reported were headache, eye dryness and burning [28]. However, this study was carried out during the COVID-19 pandemic, so the working conditions were modified. Moreover, the different questionnaires and the different terminology used to characterize the symptoms may lead to differences between studies. On the other hand, another study [19] with university students showed that the most frequent symptoms were asthenopia (13.8%) and intermittent blurred vision at distance and difficulty in focusing when looking from near to far (12.3%), similar to what was found in our study. The two questions added to our study should be considered by clinical examiners when assessing symptomatology, especially in this type of population.

Presbyopia did not affect the CISS score, which was similar in presbyopic and non-presbyopic subjects. However, the symptoms reported more frequently varied with age. The symptoms reported by older subjects are more likely to be due to presbyopia, whereas those reported by younger subjects are more likely to be due to undiagnosed and untreated accommodative and binocular dysfunctions. Furthermore, an ergonomic work environment may also lead to significant symptoms [29]. A previous study found a high prevalence of eye strain and burning in university students, which could be reduced with improved ergonomic practices [30].

Subjects who wear glasses presented a significantly higher mean score for some symptoms than those who do not wear glasses. An under- or overcorrection observed in some subjects may contribute to an increase in the symptoms in these subjects. Furthermore, although the differences found in subjects’ astigmatism compared to their habitual spectacles were not large, astigmatisms not well corrected may also lead to symptoms and influence the accommodative response [31].

A high prevalence of accommodative dysfunctions was also found, and it was higher than in previous studies of the general adult population [24,32]. The most recent study in 2020 found 21.1% of accommodative dysfunction in an adult clinical population, where 11.5% had accommodative insufficiency, 3.8% had accommodative excess and 5.8% had accommodative infacility [26]. The high visual demands required in an academic environment may justify the higher prevalence in this group of subjects. A previous study also reported changes in several accommodative parameters in university students after the university exams period, resulting in blurred vision, headache and problems with focusing [33].

The highest prevalence was found for accommodative insufficiency, similar to a previous study in a clinical population [32], which also reported this accommodative dysfunction as the most prevalent, at 11.5%.

Of the subjects who underwent an eye exam, 40% were considered symptomatic, and accommodative dysfunctions were responsible for 39.4% of the symptoms reported by them. Binocular vision dysfunctions were responsible for a small proportion of cases, and the symptoms reported by the other subjects considered symptomatic may be a result of ergonomic working conditions. These factors may also explain the lack of significant differences found in CISS scores between the subjects with and without accommodative dysfunctions, as many subjects without accommodative dysfunctions may also have had high scores for these other reasons. The similar number of hours spent in near vision may also be justified by the same reason, or they may have spent more hours in near vision in the past but reduced this time after developing an accommodative dysfunction because of the symptoms it caused. 

Another hypothesis is that they may actually spend similar amounts of time in near vision, but some of them develop accommodative dysfunctions while others do not, suggesting that the number of hours does not influence the development of accommodative dysfunctions. Instead, other factors, such as the individual crystalline lens morphology, may render some subjects more susceptible to developing accommodative dysfunctions.

The highest prevalence of accommodative dysfunctions seems to be in hyperopes. According to a previous study, accommodative dysfunctions are more frequent in subjects with uncorrected refractive errors [34]. If our sample of hyperopes were not corrected, during near-vision tasks, there would be an increased demand on their accommodative system to compensate for their refractive error. This increased demand could lead to accommodative dysfunctions, explaining this higher prevalence observed. However, our sample size of hyperopes is small and the differences found were not statistically significant, so further investigation with a larger sample, of both myopes and hyperopes, is needed to draw definitive conclusions. A recent study [35] found a significant relationship between accommodative insufficiency and moderate myopia. We did not find any evidence of an association; however, in our study, the sample size of myopes was smaller and they were not grouped by the level of refractive error.

The greatest weakness of this study is the fact that the questionnaire was shared by email among the university community, which could have disproportionately reached subjects with symptoms, resulting in a higher CISS score. In addition, some of the subjects completed the questionnaire but were not available for an eye examination, which reduced the sample size in the second part of the study.

We highlight the significance of evaluating symptomatology, accommodation, binocular vision and workplace ergonomics during clinical exams in academic populations. This population typically perform extensive near-vision tasks for prolonged periods, with substantial visual requirements, potentially leading to symptom manifestation. Regular and personalized eye examinations based on their symptomatology and the type of population are essential for detecting and addressing any vision problem properly. In addition, understanding the prevalence of associated symptoms can help healthcare professionals and educators implement appropriate interventions to reduce visual discomfort and improve the academic performance of the university population by promoting healthier visual habits and minimising the risk of visual problems. 

## Figures and Tables

**Table 1:** Criteria used to diagnose accommodative and binocular dysfunctions.

	Accommodative Dysfunctions		
	Infacility of accommodation	Excess of accommodation	Insufficiency of accommodation
Criteria	Present signs a and b	Present signs a, b and c, and two between d and f	Present signs a and b, and two between c and e
Signal	(a) Monocular accommodative facility ≤ 6 cpm and binocular ≤ 3 cpm (b) PRA ≤ 1.25 D and NRA ≤ 1.50 D	(a) Variable VA (b) Variable static retinoscopy/subjective refraction (c) Monocular accommodative facility ≤ 6 cpm with difficulty with the lens +2.00 D (d) Binocular accommodative facility ≤ 3 cpm with difficulty with the lens +2.00 D (e) MEM < +0.25 D (f) NRA ≤ 1.50 D	(a) AA at least 2.00 D below 15−0.25×age (b) Monocular accommodative facility ≤ 6 cpm with difficulty with the lens −2.00 D (c) Binocular accommodative facility ≤ 3 cpm with difficulty with the lens −2.00 D (d) MEM > +0.75 D (e) PRA ≤ 1.25 D
	Binocular dysfunctions		
	Convergence insufficiency	Convergence excess	
Criteria	Present signs a, b and c, and two of d, e, f and g	Present signs a and b, and two of c, d e and f.	
Signal	(a) Exophoria at near > 6∆ (b) PFV at near ≤ 11/14/3 (blur/break/recovery) (c) NPC > 10 cm (d) AC/A ratio < 3/1 (e) Fails BAF with +2.00 D (≤3 cpm) (f) MEM < +0.25 D (g) PRA ≤ 1.50 D	(a) Esophoria > 2∆ (b) NFV ≤ 8/16/7 (blur/break/recovery) (c) AC/A > 7/1 (d) Fails BAF with −2.00 D (≤3 cpm) (e) MEM > +0.75 D (f) PRA ≤ 1.25 D	

cpm: cycles/minute; PRA: positive relative accommodation; NRA: negative relative accommodation; VA: visual acuity; MEM: monocular estimated method; AA: amplitude of accommodation; PFV: positive fusional vergence; NFV: negative fusional vergence; NPC: near point of convergence; BAF: binocular accommodative facility.

**Table 2:** Percentage of answers for each question of the CISS survey and the two additional questions related to symptoms.

	Answer				
Question	Never	Infrequently/ Not Very Often	Sometimes	Fairly Often	Always
Eyes tired	8.3%	23.0%	41.3%	23.4%	4.0%
Uncomfortable	17.1%	27.8%	36.1%	17.9%	1.2%
Headaches	25.0%	35.3%	29.8%	2.3%	1.6%
Sleepy	23.4%	26.2%	33.7%	14.7%	2.0%
Difficulty maintaining attention/concentration	20.6%	31.7%	28.6%	16.7%	2.4%
Trouble remembering	15.5%	29.0%	34.1%	19.4%	2.0%
Double vision	58.3%	20.2%	15.9%	5.2%	0.4%
Words moving, jumping or seeming to float across the page	67.9%	22.2%	6.3%	3.6%	0.0%
Feel like reading slowly	40.9%	29.0%	21.4%	6.0%	2.8%
Eyes hurt	37.7%	28.2%	26.2%	6.3%	1.6%
Red eyes	36.5%	29.8%	23.0%	9.5%	1.2%
“Pulling” feeling around eyes	33.3%	30.6%	24.2%	11.5%	0.4%
Notice the words blurring while reading/doing close work	33.3%	31.7%	23.4%	10.3%	1.2%
Lose own place	28.2%	38.1%	24.2%	9.1%	0.4%
Need to reread the same line	15.5%	32.1%	30.6%	21.0%	0.8%
Notice the objects/words blurred at far distances	29.4%	21.8%	27.4%	16.7%	4.8%
Feel difficult to keep the objects/words clear when altering the vision from far to near and/or near to far	26.2%	30.6%	23.4%	14.7%	5.2%

**Table 3:** Prevalence of accommodative dysfunctions.

		N	%
With accommodative dysfunction		34	30.9
	Infacility of accommodation	6	5.5
	Excess of accommodation	11	10.0
	Insufficiency of accommodation	17	15.5
Without accommodative dysfunction		76	69.1
Total		110	100

**Table 4:** The mean values of phoria and negative and positive fusional vergences for far and near vision.

Far	Phoria (∆)	Positive Fusional Vergence (∆)			Negative Fusional Vergence (∆)		
		Blur	Break	Recovery	Blur	Break	Recovery
	−1.12 ± 2.33	12.85 ± 4.95	19.82 ± 6.61	9.50 ± 6.20		8.65 ± 3.45	4.51 ± 2.65
Near	Phoria (∆)	Positive Fusional Vergence (∆)			Negative Fusional Vergence (∆)		
		Blur	Break	Recovery	Blur	Break	Recovery
	−4.22 ± 4.89	20.42 ± 7.93	24.63 ± 8.32	14.63 ± 8.68	14.08 ± 5.35	20.10 ± 4.90	14.69 ± 11.39

**Table 5:** Prevalence of accommodative dysfunctions (AD) by refractive error.

	N (Percentage)				
	Without AD	With AD	Infacility	Excess	Insufficiency
Emmetropes	35 (67.3%)	17 (32.7%)	3 (5.8%)	7 (13.5%)	7 (13.5%)
Myopes	28 (80%)	7 (20%)	2 (5.7%)	0 (0.0%)	5 (14.3%)
Hyperopes	12 (57.1%)	9 (42.9%)	1 (4.8%)	4 (19%)	4 (19%)

## Data Availability

Data is contained within the article and can be accessed upon request to the Corresponding Author.
